# Identification of alternative topics to diversify medicine, dentistry, and pharmacy student theses: a mixed method study

**DOI:** 10.1186/s12909-023-04031-8

**Published:** 2023-02-13

**Authors:** Mahla Salajegheh, Somayeh Noori Hekmat, Reza Malekpour-afshar

**Affiliations:** 1 Department of Medical Education, Medical Education Development Center, Kerman University of Medical Sciences, Kerman, Iran; 2 Management and Leadership in Medical Education Research Center, Kerman University of Medical Sciences, 7616913555, Haft-Bagh Highway, Kerman, Iran; 3 Department of healthcare management, policy and economics, Faculty of management and medical information sciences, Kerman university of medical sciences, Kerman, Iran; 4 Pathology and Stem Cells Research Center, Kerman University of Medical Sciences, Kerman, Iran

**Keywords:** Academic thesis, Dentistry, Medicine, Pharmacy, Student

## Abstract

**Introduction:**

The impact of medicine, dentistry, and pharmacy student theses on public health is a crucial concern for policymakers in medical science universities. If student theses correspond to the needs of society, they can significantly affect students’ scientific and practical abilities and lead to the provision of more efficient health services. This study aimed to identify alternative topics to diversify medicine, dentistry, and pharmacy student theses.

**Methods:**

This mixed method study with an exploratory sequential design was conducted at Kerman University of Medical Science from February to June 2021. The qualitative component entailed a focus group of faculty members (*n* = 16) and students (*n* = 4) to extract alternative topics to diversify medicine, dentistry, and pharmacy student theses. The quantitative component included a questionnaire based on emerging subjects and literature review to evaluate the extracted alternative topics. Qualitative data were analyzed using conventional content analysis and quantitative data were analyzed descriptively.

**Results:**

A total of 20 key participants took part in the focus group meeting, and from 20 questionnaires, 15 were returned with a response rate of 75%. A list of 18 alternative topics was generated and five categories were identified: individual development, research, education, healthcare, and social services.

**Conclusions:**

The gap between what we know and what is seen in practice is quite large in medical and health-related professions. Alternative topics for medicine, dentistry, and pharmacy student theses contribute to turning knowledge into practice.

**Supplementary Information:**

The online version contains supplementary material available at 10.1186/s12909-023-04031-8.

## Introduction

Research is a key determinant of each country’s educational, economic, social, cultural, and industrial development. Without relying on the results of research projects, development efforts would be baseless and would not correspond with the real needs of the community [1]. Universities are the most important organizations that affect research [1, 2]. Research in medical science universities is more important due to universities’ crucial role in different aspects of public health [3]. According to Gouda et al. (2016), most students and faculty members in medical science universities consider research a useful instrument for preparing students for practice [4]. In this regard, special attention should be given to theses, which are research projects that medicine, dentistry, and pharmacy students perform during their study at the university [5].

Traditionally, a thesis serves as a capstone or summative academic and research activity and as a key component of the curriculum in medicine, dentistry, and pharmacy education. The topic students choose to pursue for their respective theses can indicate their individual interests or motivations or be aligned with a larger specific problem in their chosen field of study. In some countries, completing a thesis is not necessary for graduation from medical, dentistry, and pharmacy schools but in some medical science universities around the world, it is a mandatory part of the curriculum [6]. In Iranian medical science universities, in addition to completing their basic education, medicine, dentistry, and pharmacy students must choose a thesis topic, conduct the research under supervision of a faculty member and defend it before graduation to receive the doctorate degree [7]. Basically, the purpose of the thesis is to identify and solve one of the health system’s issues. These issues are addressed as research projects, and the theses can also address broader subjects and include educational activities, social services, technology, etc.

Medicine, dentistry, and pharmacy theses are considered important sources for production of science because they combine education and research [8]. It is expected that the results of months, even years, of the study and research of students and their collaboration with supervisors will not only discuss the health issues of the community, but also make an effort to deal with the challenges health care services are facing [9, 10].

However, the valuable goals of medical science theses have been forgotten in many settings [11]. Despite the considerable time, energy, and budget spent on conducting theses, most of their results end up in libraries or papers. As a result, large amounts of financial resources and the faculty members’ energy and time are wasted. Many results of the student theses with topics or implementation strategies that do not correspond with the needs of the society are not socially useful. Some examples are studying only the frequency distribution of a variable, the causes of a disease, the relationship between two variables, and so on, which are conducted without providing any solutions and suggestions for solving the real problems of the health system [5].

There are different reasons why some theses are not useful in addressing the common problems of public health. Students do not choose thesis topics according the real needs of the society, but base their choice on chances of being published, the time needed to complete the thesis or they choose topics based on the supervisor’s interest or in line with the supervisor’s previous research [12–15]. Similarly, Marais et al. (2019) mentioned some factors, including time and supervisory availability as two critical reasons for choosing subjects of theses among medical science students [16]. Therefore, there is a deep gap between the conducted theses and the real needs of the public health, which has caused much concern among policymakers in medical science universities.

This study aimed to identify alternative topics to diversify medicine, dentistry, and pharmacy student theses and their implementation strategies. This may lead to addressing common problems of public health during the student’s education and improving their competence in addressing these kinds of problems in their future health profession.

## Methods

### Study design

Considering the multidimensional nature of the medical science theses, which usually deal with complex human activities, they cannot be addressed from an exclusively technical perspective; therefore, a mixed-method study with an exploratory sequential design was selected. In the exploratory sequential approach, first, the researcher begins with a qualitative research phase and explores the views of participants. Then the data are analyzed, and the information is used in a second, quantitative phase. The qualitative phase may be used to build an instrument that best fits the sample under study [17].

### Setting

The study was conducted in the Education Development Center of Kerman University of Medical Science. The qualitative component of the study was conducted from February to April 2021. The quantitative component was conducted from April to June 2021.

### Literature search

We conducted non-systematic literature searches on Medline, EMBASE, Web of Science, ERIC, and Scopus with the keywords “thesis”, “alternative”, “student”, “medicine”, “dentistry”, “pharmacy”, and “dissertation” followed by additional searches of the reference lists of relevant articles. We used this literature to (i) inform the design of this study, (ii) design the guidelines for the focus group meeting and the questionnaire, and, most importantly, (iii) identify the possible alternative topics that may also be studied in other settings.

### Qualitative sampling and recruitment

For the qualitative component, we employed purposive sampling. The emphasis was on ‘purposively selecting information-rich’ cases **[**18**]**. The main inclusion criteria for the participants in the focus group were: (i) faculty members with the experience of educational activities in schools of medicine, dentistry, and pharmacy, (ii) faculty members with the experience of supervising medicine, dentistry, and pharmacy student theses, and (iii) students interested in participating in the university’s educational development affairs from schools of medicine, dentistry, and pharmacy. Participants included 16 faculty members and 4 students. They were invited by email, and then invitations were confirmed in person.

### Qualitative data collection

The focus group meeting lasted 90 minutes. In the beginning of the meeting, we gave participants a brief description of the results of the literature review. Then participants were asked the general question, “What alternative topics can be used to diversify medicine, dentistry, and pharmacy student theses?” and continued with specific questions based on the discussion. With the assistance of the facilitator, participants discussed the alternative subjects, resolved questions of clarity, and combined related alternative subjects into a single item and clarification was provided. In case of disagreement, the person who proposed the alternative topics could decide whether or not to combine it with another item. Similar alternative topics were grouped through group discussion and a label was selected for each domain. The facilitator (SN) tried to engage all participants in the discussion and gave everyone the opportunity to comment, explain, and interpret their viewpoints. All the conversations were recorded, and notes were taken from the discussions and mentioned points.

### Qualitative data analysis

The qualitative data were analyzed using the conventional content analysis method, which uses inductive content analysis [19]. The transcript was reviewed several times for better understanding and general comprehension. Then, the transcript was divided into meaning units. The meaning units were coded, and units similarly coded were interpreted for a shared meaning. MS analyzed the focus group text and drafted the manuscript. SN and RM performed a separate analysis of large parts of the text, and consensus was reached through discussion where differences in analysis appeared.

### Quantitative sampling and recruitment

The quantitative component included all the participants of the focus group.

### Quantitative data collection

Informed by the findings of the qualitative research, we developed a questionnaire with 18 items to evaluate the extracted alternative topics to diversify medicine, dentistry, and pharmacy student theses. This questionnaire evaluated each subject based on some criteria, including “leading to learning research skills,” “leading to lifelong learning,” and “leading to learning evidence-based decision-making.” Items of the questionnaire were assessed using a 5-point Likert scale ranging from strongly agree to strongly disagree, scored from 5 to 1, for statistical comparison.

Content validity was examined by computing the content validity ratio (CVR) and content validity index (CVI). The minimum acceptable CVR was 0.62 based on Lawshe’s table. The formula for calculating CVI in the Waltz and Bausell method is the number of all the respondents in “relevancy,” “clarity,” and “simplicity” criteria divided by the number of experts who gave a 3 or 4 score in the relevant question in that criterion. In this formula, if an item has a score more than 0.79, that item remains in the questionnaire. If CVI is between 0.70 and 0.79, the item needs correction and revision and if it is less than 0.70, the item is unacceptable, and it must be omitted. Face validity was explored by the expert’s comments on the simplicity of questions and their fluency. Reliability assessment was investigated by Cronbach’s alpha. Internal consistency higher than 0.7 was considered suitable.

After investigating reliability and validity, the final electronic version of the questionnaire was sent to the participants of the focus group. It was redistributed one more time at an approximately 2-week interval via E-mail and also followed up through social media.

### Quantitative data analysis

We performed a descriptive analysis using SPSS version 20.3 (i.e., mean value and standard deviation).

### Ethical approval

The Research Ethics Committee of the National Agency for Strategic Research in Medical Education approved the study (No. IR.NASRME.REC.1400.038). The participants did not receive any incentives, and participation was voluntary. Verbal consent for participation was obtained based on the proposal approved by the Ethics Committee.

## Results

### Characteristics of participants in the qualitative study component

A total of 20 participants took part in the focus group meeting. They included 16 faculty members (7 faculty members affiliated with the school of medicine, 4 affiliated with the school of dentistry, 3 affiliated with the school of pharmacy, and 2 with PhDs in medical education) and 4 students (2 medical students, 1 dentistry student, and 1 pharmacy student). The majority of participants (66.7%) were women.

### Characteristics of the participants in the quantitative study component

The final number of participants who completed the questionnaire was 16 of the 20 initially recruited, yielding a response rate of 75%. They included 12 faculty members, most of whom were from the school of medicine (64%), 23% from the school of dentistry, and 13% from the school of pharmacy. All four students completed the questionnaire. The majority of participants (70%) were women.

### Results of the literature search

Thirty-one alternative topics were extracted from the literature search. Moreover, three criteria, “leading to learning research skills”, “leading to lifelong learning”, and “leading to learning evidence-based decision-making” were extracted for the evaluation of the activities.

### Results of the qualitative component

From the 31 alternative topics that were extracted through literature search, 16 were rejected by the focus group. The focus group also suggested seven other alternative topics to diversify medicine, dentistry, and pharmacy student theses. Finally, the focus group yielded 22 alternative topics to diversify the medicine, dentistry, and pharmacy student theses. The initial alternative topics from literature review and qualitative component are included in Additional file 1 Appendix 1.

### Results of the quantitative component

The overall CVR was 0.69, which was acceptable. The CVI for all items was 0.81. Some items were corrected after face validity assessment. The Cronbach’s alpha coefficient for all items of the questionnaire was 0.80. Descriptive analysis of the questionnaire, including the mean and SD for each question, is presented in Table 1.

**Table 1:** Descriptive analysis of the questionnaire

	Alternative topics	Mean	SD
1	Participating in educational courses such as educational research, evidence-based medicine, medical education, and health system management	5.00	0.00
2	Knowledge translation of healthcare systematic reviews	5.00	0.00
3	Producing knowledge-based products, patents, software, and other technological activities	4.97	0.27
4	Publishing case series about rare diseases	4.00	0.00
5	Conducting research in the student research committee and publishing articles in Scopus, PubMed, or ISI journals	4.00	0.00
6	Conducting projects based on the product prototype and preparing a business plan	5.00	0.00
7	Voluntary service in healthcare centers for at least three months	1.72	0.70
8	Conducting post-marketing studies	5.00	0.00
9	Winning awards in educational festivals	4.93	0.25
10	Winning awards in the Educational Innovative Ideas Festival	5.00	0.00
11	Winning medals (gold, silver, or bronze) in Students Scientific Olympiads of medical science	4.00	0.00
12	Voluntary service in doctor’s offices, clinics, and healthcare centers for at least three months	4.97	0.27
13	Participating as a research assistant in PhD dissertations	1.62	0.71
14	Voluntary services in knowledge-based companies for at least three months	5.00	0.00
15	Participating as a research assistant in residency dissertations	1.43	0.62
16	Participating as a research assistant in residency dissertations	5.00	0.00
17	Voluntary services in nursing homes for at least three months	4.00	0.00
18	Voluntary service in drugstores affiliated with the university for at least three months	4.97	0.27
19	Conducting a research plan in one of the research centers of the university	5.00	0.00
20	Conducting group research with interdisciplinary topics	4.93	0.25
21	Voluntary service in non-governmental organizations or health charities for at least three months	5.00	0.00
22	Voluntary service in clinics for at least three months	1.56	0.72

According to the results of the questionnaire analysis, 18 alternative topics were eligible to diversify medicine, dentistry, and pharmacy student theses. They were classified into five domains, including individual development, research, education, healthcare, and social services. The individual development domain focuses on developing competencies in the medical science student as a future health professional. The research domain represents educational research activities, including involvement in projects that share new knowledge, pursuing the students’ interests, and translating the research findings to respond to the public health needs. The domain of education highlights the competencies in the teaching and learning process, including various teaching and student assessment methods. The healthcare domain refers to the role of medical science students in local community health. The social services domain represents exposures that enhance the student’ competencies in social skills that impact patient care. The alternative topics to diversify medicine, dentistry, and pharmacy student theses are presented in Table 2.

**Table 2:** The alternative topics to diversify medicine, dentistry, and pharmacy student theses

Domain	Alternative topics
Individual development	1. Participating in educational courses such as educational research, evidence-based medicine, medical education, and health system management
Research	2. Translating the knowledge of healthcare systematic reviews
	3. Producing knowledge-based products, patents, software, and other technological activities
	4. Publishing case series about rare diseases
	5. Participating as a research assistant in PhD or residency dissertations
	6. Conducting research in the student research committee and publishing the articles in Scopus, PubMed, or ISI journals
	7. Conducting a research plan in one of the research centers of the university
	8. Conducting projects based on the product prototype and preparing business plans
	9. Conducting post-marketing studies
	10. Conducting group research with interdisciplinary topics
Education	11. Winning awards in educational festivals
	12. Winning awards in the Educational Innovative Ideas Festival
	13. Winning medals (gold, silver, or bronze) in Scientific Olympiads of medical science students
Healthcare	14. Voluntary service in doctor’s offices, clinics, and healthcare centers for at least three months
	15. Voluntary service in drugstores affiliated with the university for at least three months
Social services	16. Voluntary service in non-governmental organizations or health charities for at least three months
	17. Voluntary services in nursing homes for at least three months
	18. Voluntary services in knowledge-based companies for at least three months

## Discussion

To the best of our knowledge, our study is the first to identify alternative topics to diversify medicine, dentistry, and pharmacy student theses. The study identified 18 alternative subjects to diversify medicine, dentistry, and pharmacy student theses, classified into five domains, including individual development, research, education, healthcare, and social services.

Although some competencies such as educational research, evidence-based medicine, medical education, and health system management are essential for a medical science student as a future researcher/practitioner/teacher, most of these students lack extracurricular skills. We found that “Individual development” was an alternative topic domain to diversify medicine, dentistry, and pharmacy student theses. The mentioned competencies provide important benefits to the educational mission of the medical science universities by supporting the professional identity of medical science students and are essential to the continued development of medical education as a discipline. In addition, these extracurricular skills assist students to identify the problems related to public health and find relevant evidence to solve them in the future. Andrew Jay et al. (2013) explored the effects of a program which addressed the core teaching competencies of medical students for further teaching roles through an anatomy program. Their findings indicated that the students achieved core competencies of a medical educator and felt prepared for the teaching demands of residency [20]. Similarly, Alahdab et al. (2012) evaluated an educational program of evidence-based medicine performed for 50 undergraduate medical students. They reported a statistically significant increase in medical students’ perceived ability to go through evidence-based medicine and suggested developing the proper environment to facilitate transforming current medical education and practice to an evidence-based standard [21].

Most of the alternative topics identified to diversify medicine, dentistry, and pharmacy student theses were in the “Research” domain. Potentially, research activities provide clues for the identification of possible experts since effective research experiences during medical science universities predict future career achievements in academic medicine [22]. Different research projects may provide additional opportunities for medical science students to pursue their own interests and translate the research findings to address public health needs. Shahiwala (2017) provided a project-based business-proposal-building activity and a pharmacy management course for pharmacy students. The results showed that the project helped the students understand management concepts more clearly and increased their interest and confidence to start a pharmacy [23]. Hansen et al. (2022) implemented and evaluated a program to provide interdisciplinary education to nursing and medical students and clinical faculty about facilitating end of life care. They reported that the team meetings and the interaction between team members benefited the patients and their families [24]. Moreover, it is worth mentioning that even though research is the most common choice for theses, our results reflect that besides research, there are other possibilities that can be considered as theses for medical science students.

We found that “Education” was one of the main domains of alternative topics to diversify medicine, dentistry, and pharmacy student theses. This is consistent with the aims and expected learning outcomes of all medical science students. However, we found some new and important educational alternative topics in relation to the increased motivation and effort of students to become experts in medical education. This finding highlights the high interest that is demonstrated by some medical science students in health education. In this regard, Hajinezhad et al. (2021) explain that the national scientific Olympiad for medical science students may be an effective opportunity to motivate students to acquire educational skills, enhance clinical reasoning, problem-solving skills, and team work and eventually lead to improvements in the health system [25]. Therefore, these student Olympiads and competitions, as they are implemented in Iran, are quite similar to student theses. The aims of these competitions include promoting creativity, innovation, and critical thinking in students, encouraging teamwork, strengthening interdisciplinary activities, encouraging inter-university scientific exchange, and trying to improve students’ ability to criticize and solve health system problems in a friendly, moral, and lively atmosphere. The students have to work for a period of at least 1 year to succeed in these competitions. In this process, they gain valuable competencies that will help them contribute to social health in future.

The “Healthcare” domain includes some of the important alternative subjects to diversify medicine, dentistry, and pharmacy student theses. The subjects in this domain include voluntary service in doctor’s offices, clinics, healthcare centers, or drugstores affiliated with the university. Rockey et al. (2021) explained that medical student-run health clinics offer numerous services to patients and are also noteworthy phenomena in medical education. They recommended that educational policy-makers pay more attention to issues of health and the medical society, considering the role that students can play in addressing local healthcare needs [26]. Considering students’ interest and professional career pathways in the future, our findings suggest that it is necessary to provide opportunities for them to practice while they are pursuing their degree in university. For example, some students may decide to pursue their professional careers as clinic doctors, family doctors, or pharmacists in pharmacies. Therefore, performing an alternative thesis in the “Healthcare” domain addresses their needs and improves the skills necessary for their future careers.

An interesting finding of our study is the alternative topics in the “Social services” domain, which includes voluntary services in non-governmental organizations, health charities, nursing homes, or knowledge-based companies. Exposure to these settings gives students practical understanding of social factors that impact patient care [27]. Khasanzyanova (2017) stated that volunteering with non-governmental organizations helps acquire soft skills, which are essential to complement professional skills and expertise [28]. Hu et al. (2022) explored the changes in rural health care because of voluntarily service-learning programs of medical students in Taiwan. They found that these programs benefited medical students by providing an opportunity for transferring their theoretical knowledge to the clinical setting and benefited the community by balancing the rural health care supply and demand. They suggested implementing similar health care service learning programs within other contexts [29]. Also, Tinker et al. (2017) investigated the influence that volunteering with older people in a care home has on the medical students’ perceptions of older people. They reported achieving a better understanding of older people and gaining transferable skills related to communicating with them, especially those with cognitive impairment [30].

The findings of our study found that the alternative topics identified to diversify medicine, dentistry, and pharmacy student theses impact not only the students, but also public health as a whole. There is need for a broader approach to implement these topics and evaluate their impact on students and their social accountability in future research.

Some limitations of this study should be considered. The research was conducted in one university with a limited number of key participants; therefore, results may not be generalizable to other contexts. However, recruiting different participants, including faculty members and students led to the development of a list of alternative topics. Further research at other medical science universities is recommended to determine if there are other important alternative topics not identified in this study and if these results are generalizable to other universities. Another limitation was the relatively limited literature on alternative topics to diversify medicine, dentistry, and pharmacy student theses. There is an obvious scarcity of this type of research, so future research is suggested.

## Conclusions

Identification of alternative topics to diversify medicine, dentistry, and pharmacy student theses provides insight into the impact of these activities on public health development. The findings can be used in other settings to contribute to social health services. Some of them may need revision based on the specific aspects of each context.

## Supplementary Information

**Additional file 1.** The initial alternative subjects from literature review and qualitative component.

## Data Availability

The datasets used and/or analyzed in the current study are available from the corresponding author on reasonable request.
