# Supplementary material for: Identification of alternative topics to diversify medicine, dentistry, and pharmacy student theses: a mixed method study
Source: BMC Med Educ. 2023 Feb 13;23:110. doi: 10.1186/s12909-023-04031-8 (PMC9923902; doi:10.1186/s12909-023-04031-8)
Supplement: Supplementary file 1 — Additional file 1. The initial alternative subjects from literature review and qualitative component. [file 12909_2023_4031_MOESM1_ESM.docx]

The initial alternative subjects from literature review and qualitative component

| Phase of study | Alternative subjects | Decision |
| --- | --- | --- |
| Literature search | 1. Participating in educational courses such as educational research, evidence-based medicine, medical education, health system management | Eligible |
|  | 1. Writing and publishing review articles | Not eligible |
|  | 1. Knowledge translation of healthcare systematic reviews | Eligible |
|  | 1. Converting 3 dissertations from previous years into articles in compliance with ethical principles and intellectual property rights | Not eligible |
|  | 1. Producing knowledge-based product, patent, software, and other technological activities | Eligible |
|  | 1. Publishing case series about rare diseases | Eligible |
|  | 1. Writing proposals to attract research funds from institutions outside the university | Not eligible |
|  | 1. Voluntary service in research development centers of the university for 6 months to help improve research | Not eligible |
|  | 1. Voluntary service in journals of the university for 6 months to help improve the quality of publications | Not eligible |
|  | 1. Conducting a research in the student research committee and publishing article in Scopus, PubMed, or ISI journals | Eligible |
|  | 1. Collaboration as a researcher assistant in a research in the student research committee | Not eligible |
|  | 1. Conducting projects based on the product prototype and preparing business plan | Eligible |
|  | 1. Cooperation in drug economics studies under the supervision of a faculty member | Not eligible |
|  | 1. Conducting post marketing studies | Eligible |
|  | 1. Conducting interdisciplinary thesis | Not eligible |
|  | 1. Winning award in educational festivals | Eligible |
|  | 1. Winning award in Educational Innovative Ideas Festival | Eligible |
|  | 1. Winning a medal (gold or silver or bronze) in Scientific Olympiad of medical sciences students | Eligible |
|  | 1. Collaboration in writing and publication of reference books | Not eligible |
|  | 1. Collaboration in producing educational virtual contents | Not eligible |
|  | 1. Being as a teacher assistant according to the field of study | Not eligible |
|  | 1. Compilation of booklets in a way that has innovation and is based on scientific evidence | Not eligible |
|  | 1. Voluntary service in doctor's offices for at least three months | Eligible |
|  | 1. Compilation of clinical guidelines for general physicians | Not eligible |
|  | 1. Voluntary service in health camps | Not eligible |
|  | 1. Voluntary service in health centers for 6 months | Not eligible |
|  | 1. Voluntary service in non-governmental organizations for at least three months | Eligible |
|  | 1. Voluntary services in nursing homes for at least three months | Eligible |
|  | 1. Voluntary service in drugstores affiliated to university for at least three months | Eligible |
|  | 1. Voluntary services in knowledge-based companies for at least three months | Eligible |
|  | 1. Voluntary services in a specific scientific/skill field or social activities related to health for at least 6 months | Not eligible |
| Qualitative component | 1. Conducting a research plan in one of the research centers of the university | Eligible |
|  | 1. Conducting group researches with interdisciplinary subjects | Eligible |
|  | 1. Voluntary service in health charities for at least three months | Eligible |
|  | 1. Voluntary service in clinics for at least three months | Eligible |
|  | 1. Voluntary service in healthcare centers for at least three months | Eligible |
|  | 1. Participating as a research assistant in PhD dissertation | Eligible |
|  | 1. Participating as a research assistant in residency dissertation | Eligible |
